# Impact of sociodemographic status of countries on neonatal analgosedation management

**DOI:** 10.1038/s41390-024-03262-9

**Published:** 2024-05-22

**Authors:** Ursula Felderhoff-Müser

**Affiliations:** 1 Department of Paediatrics I, Neonatology, Paed. Intensive Care, Paed. Infectiology, Paed. Neurology, University Hospital Essen, University of Duisburg-Essen, Essen, Germany; 2 Center of Translational Neuro- and Behavioral Sciences, C-TNBS, Faculty of Medicine, University of Duisburg- Essen, Essen, Germany

Despite advances in neonatal care a significant proportion of infants requires hospitalisation shortly after birth due to prematurity, congenital malformations or other severe illness. Live-saving neonatal intensive care is frequently associated with distress, painful procedures and post-procedural pain, which significantly impact short- and long-term outcomes of these children.^1^ NICU treatment overlaps with a period of rapid brain maturation and high vulnerability to pain and distress as shown for example by alterations of brain connectivity impacting neurological development in very preterm infants.^2^

Recognizing infants in distress and pain is a quality indicator for good clinical practise, however it requires high professional skills, and also high amounts of trained staff. In low- and middle- income countries the number of preterm or sick born infants receiving neonatal intensive care treatment is largely increasing as more NICUs, technical equipment and trained staff is available. These NICUS often work with limited knowledge and restricted human and technical resources resulting in high complication rates.^3^ However, a variety of studies even from non-resource-limited countries have reported a lack of or inconsistent analgosedation during painful procedures and missing treatment protocols and guidelines. Furthermore, the choice of pain scales, medications, and dosages considerably vary among NICUs in different European countries. The use of pain assessment tools also largely depends on nurses training programs and their initiative within the team.^4,5^

In the article by Arribas et al. in *Pediatric Research* (2024 Feb 13. 10.1038/s41390-024-03032-7.), the authors, a group of well-known experts in the field of neonatal pain research, set out to collect information on the use of analgesics and sedatives and management of neonatal pain and distress in a global, prospective, cross-sectional study. 28 questions were developed and consented in a Delphi process among authors and distributed via the Survey Monkey Platform to Paediatric Societies, working Groups and neonatal social networks. This world-wide survey concentrated on painful procedures and interventions including therapeutic hypothermia and the use of pharmacological agents grouped by action such as opioids, hypnotics, analgesics, muscle relaxants and non-opioid analgesics (paracetamol, acetaminophen). 1304 responses from 98 countries were collected, most of them from Europe related to the authors professional networks. In addition, socioeconomic differences were established using the Sociodemographic Index, a composite average of rankings of incomes per capita, average educational attainment, and fertility rates of all areas in the Global Burden of Disease Project. This index has previously been successfully used in a variety of studies on the incidence and mortality from prematurity and perinatal asphyxia (https://ghdx.healthdata.org/record/ihme-data/gbd-2019-socio-demographic-index-sdi-1950-2019).

This first global survey largely extends our knowledge on the various neonatal analgosedation practices. These not only widely depend on the region or country but also on the Sociodemographic Index. Overall, only 60% of NICUs reported on the use of analgosedation guidelines and only one third of responders routinely used neonatal pain scales, mostly PIPP, PIPP-R, NPASS, NIPS and CRIES, in more than 80% of their patients with a tendency towards greater attention to the implementation of standardised pain assessment and management in high-resource settings.

Increasing evidence suggests that sensory stimuli applied during a painful procedure can reduce behavioral responses to pain. These include sensorimotor stimuli such as i.e. swaddling, facilitated tucking, rocking, (parental) touch, maternal/parental voice, massage, sucking, application of sucrose/glucose, music therapy and reduction of bright light. Unfortunately, the use of those non-pharmacological methods was not included in the survey possibly given the uncertainty of evidence.^6^

Countries with high Sociodemographic Index were more likely to have guidelines in place and showed a higher adherence to protocols. However, a variety of different medications was in use in different settings, some of them not recommended for the patient group treated, such as the broad use of midazolam for preterm infants < 32 weeks gestation. In addition to differences in the use of structured analgosedation management dependent on SDI, different strategies were in place for procedures such as i.e. intubation, invasive ventilation and therapeutic hypothermia. Although overall response rates differed between countries, with higher rates in countries with higher Sociodemographic Index, this study clearly shows the large need for introducing and/or unifying protocols for pain assessment and management of analgosedation.

In addition to the structured distribution of guidelines via local neonatal societies, these findings and circumstances in middle- and low-resource settings call for an urgent need for multidisciplinary education programs, adapted to the specificities of each region. Awareness for pain for the structured use of pain scales not only for nurses, but also for physicians needs to be raised. Not only in regions with restricted resources, parents should have a special role to play in the assessment and also non-pharmacological management of painful situations, in addition to the fact that their presence may positively influence their infant’s distress. Identification of novel and/or multiple pain biomarkers such as heart rate, heart rate variability (i.e. NIPE), respiratory rate, oxygen saturation and skin conductance may offer potential opportunities for objective interdisciplinary pain management in the NICU.^7^ In the future, artificial intelligence (AI) tools such as machine learning from nurse validated Neonatal Facial Coding System (NFCS) data might optimize pain assessment, without increasing human resources.^8^ Furthermore, pre-clinical and clinical research efforts should be directed towards optimizing use, depth and duration of sedation and analgesia protocols, preferably in combination with potentially neuroprotective agents such as dexmedetomidine, also taking (additional) non-pharmacological methods into consideration.
